# 4,4′-[(2,7-Dibromo­fluorene-9,9-di­yl)dimethyl­ene]dipyridinium bis­(perchlorate)

**DOI:** 10.1107/S1600536810021859

**Published:** 2010-06-18

**Authors:** Zongwei Xuan, Shanshan Zhao, Lude Lu, Xin Wang, Xujie Yang

**Affiliations:** a Materials Chemistry Laboratory, Nanjing University of Science and Technology, Nanjing 210094, People’s Republic of China; b New Materials & Function Coordination Chemistry Laboratory, Qingdao University of Science and Technology, Qingdao Shandong 266042, People’s Republic of China

## Abstract

In the crystal of the title compound, C_25_H_20_Br_2_N_2_^2+^·2ClO_4_^−^, inter­molecular N—H⋯O and C—H⋯O hydrogen bonds, along with C—H⋯π inter­actions, stabilize the crystal structure.

## Related literature

A variety of ligands of different mol­ecular dimensions and functional properties have been utilized in the preparation of numerous supra­molecular assemblies with exotic architectures, see: Applegarth *et al.*, (2005 ▶). For related structures, see: Meerssche *et al.* (1979 ▶, 1980 ▶).
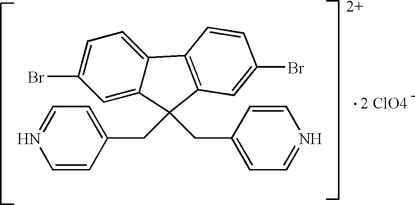


## Experimental

### 

#### Crystal data

C_25_H_20_Br_2_N_2_^2+^·2ClO_4_^−^
*M* *_r_* = 707.15
Monoclinic, 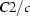

*a* = 15.605 (3) Å
*b* = 11.267 (2) Å
*c* = 16.318 (3) Å
β = 117.60 (3)°
*V* = 2542.6 (11) Å^3^
*Z* = 4
Mo *K*α radiation
μ = 3.45 mm^−1^
*T* = 295 K
0.25 × 0.20 × 0.18 mm

#### Data collection

Enraf–Nonius CAD-4 diffractometer
Absorption correction: ψ scan (North *et al.*, 1968 ▶) *T*_min_ = 0.441, *T*_max_ = 0.537
11835 measured reflections
2915 independent reflections
2611 reflections with *I* > 2σ(*I*)
*R*_int_ = 0.062
3 standard reflections every 100 reflections  intensity decay: none

#### Refinement

*R*[*F* ^2^ > 2σ(*F* ^2^)] = 0.039
*wR*(*F* ^2^) = 0.103
*S* = 1.06
2915 reflections
177 parameters
H-atom parameters constrained
Δρ_max_ = 1.01 e Å^−3^
Δρ_min_ = −0.79 e Å^−3^

### 

Data collection: *CAD-4 Software* (Enraf–Nonius, 1989 ▶); cell refinement: *CAD-4 Software*; data reduction: *NRCVAX* (Gabe *et al.*, 1989 ▶); program(s) used to solve structure: *SHELXS97* (Sheldrick, 2008 ▶); program(s) used to refine structure: *SHELXL97* (Sheldrick, 2008 ▶); molecular graphics: *SHELXTL/PC* (Sheldrick, 2008 ▶); software used to prepare material for publication: *WinGX* (Farrugia, 1999 ▶).

## Supplementary Material

Crystal structure: contains datablocks global, I. DOI: 10.1107/S1600536810021859/hg2685sup1.cif

Structure factors: contains datablocks I. DOI: 10.1107/S1600536810021859/hg2685Isup2.hkl

Additional supplementary materials:  crystallographic information; 3D view; checkCIF report

## Figures and Tables

**Table 1:** Hydrogen-bond geometry (Å, °) *Cg*3 is the centroid of the C1–C6 ring.

*D*—H⋯*A*	*D*—H	H⋯*A*	*D*⋯*A*	*D*—H⋯*A*
N1—H1*A*⋯O3	0.86	2.24	2.997 (3)	148
C11—H11*A*⋯O1	0.93	2.57	3.196 (3)	125
C12—H12*A*⋯O4^i^	0.93	2.44	3.193 (3)	138
C13—H13*A*⋯O1^ii^	0.93	2.47	3.376 (3)	164
C10—H10*A*⋯*Cg*3	0.93	2.93	3.688 (2)	140

Symmetry codes: (i) 
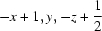
; (ii) 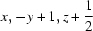.

**Fig. 1.**
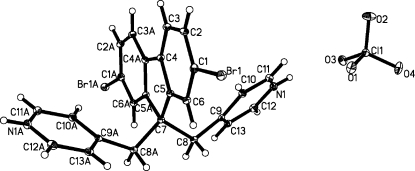
The structure of the title compound showing 30% probability displacement ellipsoids and the atom-numbering scheme.

## supplementary crystallographic information

### Comment

A variety of ligands of different molecular dimensions and functional properties were utilized for the preparation of numerous supramolecular assemblies of exotic architectures as reported in the recent literature (Applegarth *et al.*, 2005). Herein, we report a new bipyridine derivative of 2,7-dibromo-9,9-(4-pyridyl-methyl) fluorene [DBPMF].

scheme I

The structure of the title compound contains a protonated 2,7-dibromo-9,9-bis(4-pyridinium-methyl) fluorene dications DBPMFH_2_^2+^ and two perchlorate anions ClO_4_^-^. All the bond lengths and bond angles in the phenyl ring and five-membered ring are corresponding with those observed in 2-acetylaminofluorene (Meerssche *et al.*, 1980) and 4-acetylamino-fluorene (Meerssche *et al.*, 1979). Two bromine atoms along with the thirteen atoms of fluorenyl ring are coplanar (P1) and the biggest deviation is 0.038Å for C6 atom. The dihedral angle between the plane P1 and the pyridyl ring containing N1 atom is 72.11 (2)°.

In the crystal lattice, there are four types of supramolecular interactions (Table 1), including N—H···O hydrogen bonds, C—H···O potential hydrogen bonds, C—H···π supramolecular interaction and π–π stacking interactions. Among these supramolecular interactions, the two types N—H···O hydrogen bonds link two DBPMFH_2_^2+^ cations with two ClO_4_^-^ anions to construct one-dimensional chains, then the other supramolecular interactions help the 1D chains to form three-dimensional net-works, which stabilize the crystal structure.

### Experimental

DBPMF was synthesized by the reaction of 2,7-dibromofluorene (3.24 g, 0.01 mol) and 4-chloromethyl pyridine hydrochloride (1.64 g, 0.02 mol) in DMSO (70 ml). The title compound was obtained by the reaction of DBPMF (2.55 g, 5.0 mmol) and HClO_4_ (0.26 ml, 5.0 mmol) in EtOH (50 ml). Single crystals suitable for x-ray measurements were obtained by recrystallization at room temperature.

### Refinement

H atoms were fixed geometrically and allowed to ride on their attached atoms, with C—H distances=0.93-0.97 Å, N—H distance=0.86Å and with U_iso_=1.2-1.5U_eq_.

### Crystal data

**Table:**

C_25_H_20_Br_2_N_2_^2+^·2ClO_4_^−^	*F*(000) = 1408
*M**_r_* = 707.15	*D*_x_ = 1.847 Mg m^−^^3^
Monoclinic, *C*2/*c*	Mo *K*α radiation, λ = 0.71073 Å
Hall symbol: -C 2yc	Cell parameters from 25 reflections
*a* = 15.605 (3) Å	θ = 4–14°
*b* = 11.267 (2) Å	µ = 3.45 mm^−^^1^
*c* = 16.318 (3) Å	*T* = 295 K
β = 117.60 (3)°	Block, yellow
*V* = 2542.6 (11) Å^3^	0.25 × 0.20 × 0.18 mm
*Z* = 4	

### Data collection

**Table:**

Enraf–Nonius CAD-4 diffractometer	2611 reflections with *I* > 2σ(*I*)
Radiation source: fine-focus sealed tube	*R*_int_ = 0.062
graphite	θ_max_ = 27.5°, θ_min_ = 3.1°
ω/2θ scans	*h* = −20→20
Absorption correction: ψ scan (North *et al.*, 1968)	*k* = −14→14
*T*_min_ = 0.441, *T*_max_ = 0.537	*l* = −21→21
11835 measured reflections	3 standard reflections every 100 reflections
2915 independent reflections	intensity decay: none

### Refinement

**Table:**

Refinement on *F*^2^	Primary atom site location: structure-invariant direct methods
Least-squares matrix: full	Secondary atom site location: difference Fourier map
*R*[*F*^2^ > 2σ(*F*^2^)] = 0.039	Hydrogen site location: inferred from neighbouring sites
*wR*(*F*^2^) = 0.103	H-atom parameters constrained
*S* = 1.06	*w* = 1/[σ^2^(*F*_o_^2^) + (0.0681*P*)^2^ + 0.5103*P*] where *P* = (*F*_o_^2^ + 2*F*_c_^2^)/3
2915 reflections	(Δ/σ)_max_ = 0.001
177 parameters	Δρ_max_ = 1.01 e Å^−^^3^
0 restraints	Δρ_min_ = −0.79 e Å^−^^3^

### Special details

**Table:**

Geometry. All esds (except the esd in the dihedral angle between two l.s. planes) are estimated using the full covariance matrix. The cell esds are taken into account individually in the estimation of esds in distances, angles and torsion angles; correlations between esds in cell parameters are only used when they are defined by crystal symmetry. An approximate (isotropic) treatment of cell esds is used for estimating esds involving l.s. planes.
Refinement. Refinement of *F*^2^ against ALL reflections. The weighted *R*-factor wR and goodness of fit *S* are based on *F*^2^, conventional *R*-factors *R* are based on *F*, with *F* set to zero for negative *F*^2^. The threshold expression of *F*^2^ > σ(*F*^2^) is used only for calculating *R*-factors(gt) etc. and is not relevant to the choice of reflections for refinement. *R*-factors based on *F*^2^ are statistically about twice as large as those based on *F*, and *R*- factors based on ALL data will be even larger.

### Fractional atomic coordinates and isotropic or equivalent isotropic displacement parameters (Å^2^)

**Table:**

	*x*	*y*	*z*	*U*_iso_*/*U*_eq_	
Br1	−0.201221 (15)	0.319251 (18)	−0.114789 (15)	0.01844 (12)	
N1	0.33006 (13)	0.39368 (16)	0.24329 (14)	0.0178 (4)	
H1A	0.3819	0.3707	0.2418	0.021*	
C1	−0.13197 (15)	0.2901 (2)	0.01439 (16)	0.0153 (4)	
C2	−0.11455 (16)	0.17300 (17)	0.04368 (17)	0.0162 (5)	
H2A	−0.1377	0.1115	0.0009	0.019*	
C3	−0.06207 (15)	0.14838 (19)	0.13788 (15)	0.0154 (4)	
H3A	−0.0500	0.0704	0.1589	0.019*	
C4	−0.02836 (14)	0.24228 (18)	0.19940 (15)	0.0140 (4)	
C5	−0.04692 (14)	0.36049 (18)	0.16834 (16)	0.0141 (4)	
C6	−0.09989 (14)	0.38606 (18)	0.07490 (15)	0.0146 (4)	
H6A	−0.1134	0.4638	0.0536	0.017*	
C7	0.0000	0.4457 (2)	0.2500	0.0123 (5)	
C8	0.07543 (14)	0.52981 (17)	0.24235 (15)	0.0136 (4)	
H8A	0.0429	0.5737	0.1849	0.016*	
H8B	0.0965	0.5870	0.2924	0.016*	
C9	0.16421 (14)	0.47237 (18)	0.24518 (15)	0.0133 (4)	
C10	0.16000 (15)	0.39694 (18)	0.17545 (15)	0.0157 (4)	
H10A	0.1004	0.3722	0.1290	0.019*	
C11	0.24415 (15)	0.35912 (19)	0.17540 (16)	0.0175 (4)	
H11A	0.2413	0.3098	0.1285	0.021*	
C12	0.33797 (15)	0.46320 (19)	0.31370 (16)	0.0183 (4)	
H12A	0.3986	0.4841	0.3605	0.022*	
C13	0.25553 (15)	0.50303 (18)	0.31584 (15)	0.0152 (4)	
H13A	0.2606	0.5505	0.3645	0.018*	
Cl1	0.41022 (4)	0.31707 (4)	0.05644 (4)	0.01488 (15)	
O1	0.31184 (11)	0.35890 (17)	0.01812 (12)	0.0258 (4)	
O2	0.41264 (16)	0.19359 (15)	0.03767 (15)	0.0321 (5)	
O3	0.45717 (12)	0.33505 (14)	0.15648 (12)	0.0213 (4)	
O4	0.46088 (12)	0.38494 (16)	0.01772 (12)	0.0286 (4)	

### Atomic displacement parameters (Å^2^)

**Table:**

	*U*^11^	*U*^22^	*U*^33^	*U*^12^	*U*^13^	*U*^23^
Br1	0.02330 (17)	0.01761 (16)	0.01334 (17)	−0.00066 (7)	0.00760 (12)	−0.00009 (7)
N1	0.0150 (8)	0.0174 (9)	0.0236 (10)	0.0018 (7)	0.0112 (8)	0.0021 (8)
C1	0.0146 (9)	0.0182 (9)	0.0130 (10)	−0.0009 (8)	0.0064 (8)	0.0001 (9)
C2	0.0211 (11)	0.0122 (10)	0.0171 (12)	−0.0033 (7)	0.0105 (10)	−0.0052 (8)
C3	0.0200 (10)	0.0111 (9)	0.0167 (11)	0.0006 (8)	0.0097 (9)	0.0000 (9)
C4	0.0152 (9)	0.0124 (9)	0.0165 (11)	0.0002 (7)	0.0090 (8)	0.0018 (8)
C5	0.0144 (9)	0.0106 (9)	0.0196 (11)	−0.0009 (7)	0.0097 (8)	−0.0020 (9)
C6	0.0158 (9)	0.0119 (9)	0.0171 (10)	−0.0002 (7)	0.0086 (8)	0.0008 (8)
C7	0.0125 (12)	0.0115 (13)	0.0135 (14)	0.000	0.0064 (11)	0.000
C8	0.0160 (9)	0.0095 (8)	0.0167 (10)	−0.0007 (7)	0.0088 (8)	0.0001 (8)
C9	0.0162 (9)	0.0108 (9)	0.0152 (10)	−0.0005 (7)	0.0091 (8)	0.0032 (8)
C10	0.0165 (9)	0.0158 (10)	0.0154 (10)	−0.0007 (8)	0.0079 (8)	0.0000 (8)
C11	0.0207 (10)	0.0148 (10)	0.0201 (12)	0.0005 (8)	0.0121 (9)	0.0000 (9)
C12	0.0153 (9)	0.0183 (10)	0.0184 (11)	−0.0014 (8)	0.0054 (8)	0.0019 (9)
C13	0.0181 (10)	0.0134 (9)	0.0135 (10)	−0.0010 (8)	0.0068 (8)	0.0017 (8)
Cl1	0.0145 (3)	0.0161 (3)	0.0132 (3)	−0.00181 (16)	0.0057 (2)	−0.00099 (17)
O1	0.0162 (8)	0.0345 (9)	0.0247 (9)	0.0037 (7)	0.0077 (7)	0.0074 (8)
O2	0.0390 (10)	0.0178 (9)	0.0283 (11)	−0.0006 (7)	0.0061 (9)	−0.0061 (7)
O3	0.0210 (8)	0.0273 (8)	0.0128 (8)	−0.0003 (6)	0.0055 (7)	−0.0034 (7)
O4	0.0253 (8)	0.0390 (10)	0.0264 (9)	−0.0070 (7)	0.0161 (7)	0.0037 (8)

### Geometric parameters (Å, °)

**Table:**

Br1—C1	1.900 (2)	C7—C8	1.561 (2)
N1—C11	1.342 (3)	C8—C9	1.510 (3)
N1—C12	1.348 (3)	C8—H8A	0.9700
N1—H1A	0.8600	C8—H8B	0.9700
C1—C2	1.387 (3)	C9—C10	1.397 (3)
C1—C6	1.392 (3)	C9—C13	1.399 (3)
C2—C3	1.394 (3)	C10—C11	1.381 (3)
C2—H2A	0.9300	C10—H10A	0.9300
C3—C4	1.384 (3)	C11—H11A	0.9300
C3—H3A	0.9300	C12—C13	1.378 (3)
C4—C5	1.407 (3)	C12—H12A	0.9300
C4—C4^i^	1.468 (4)	C13—H13A	0.9300
C5—C6	1.387 (3)	Cl1—O2	1.4289 (18)
C5—C7	1.526 (3)	Cl1—O4	1.4395 (17)
C6—H6A	0.9300	Cl1—O1	1.4423 (16)
C7—C5^i^	1.526 (3)	Cl1—O3	1.4609 (19)
C7—C8^i^	1.561 (2)		
			
C11—N1—C12	122.30 (19)	C9—C8—C7	116.89 (17)
C11—N1—H1A	118.9	C9—C8—H8A	108.1
C12—N1—H1A	118.9	C7—C8—H8A	108.1
C2—C1—C6	123.1 (2)	C9—C8—H8B	108.1
C2—C1—Br1	117.84 (18)	C7—C8—H8B	108.1
C6—C1—Br1	119.06 (17)	H8A—C8—H8B	107.3
C1—C2—C3	119.4 (2)	C10—C9—C13	117.85 (19)
C1—C2—H2A	120.3	C10—C9—C8	122.64 (18)
C3—C2—H2A	120.3	C13—C9—C8	119.28 (19)
C4—C3—C2	118.7 (2)	C11—C10—C9	120.1 (2)
C4—C3—H3A	120.7	C11—C10—H10A	119.9
C2—C3—H3A	120.7	C9—C10—H10A	119.9
C3—C4—C5	121.1 (2)	N1—C11—C10	119.8 (2)
C3—C4—C4^i^	130.12 (13)	N1—C11—H11A	120.1
C5—C4—C4^i^	108.75 (13)	C10—C11—H11A	120.1
C6—C5—C4	120.7 (2)	N1—C12—C13	119.5 (2)
C6—C5—C7	129.05 (19)	N1—C12—H12A	120.2
C4—C5—C7	110.21 (19)	C13—C12—H12A	120.2
C5—C6—C1	117.00 (19)	C12—C13—C9	120.3 (2)
C5—C6—H6A	121.5	C12—C13—H13A	119.8
C1—C6—H6A	121.5	C9—C13—H13A	119.8
C5—C7—C5^i^	102.1 (2)	O2—Cl1—O4	110.40 (13)
C5—C7—C8^i^	112.17 (11)	O2—Cl1—O1	110.72 (12)
C5^i^—C7—C8^i^	112.75 (11)	O4—Cl1—O1	109.07 (11)
C5—C7—C8	112.75 (11)	O2—Cl1—O3	108.98 (11)
C5^i^—C7—C8	112.17 (11)	O4—Cl1—O3	108.89 (10)
C8^i^—C7—C8	105.2 (2)	O1—Cl1—O3	108.74 (11)

Symmetry codes: (i) −*x*, *y*, −*z*+1/2.

### Hydrogen-bond geometry (Å, °)

**Table:**

Cg3 is the centroid of the C1–C6 ring.

**Table:**

*D*—H···*A*	*D*—H	H···*A*	*D*···*A*	*D*—H···*A*
N1—H1A···O3	0.86	2.24	2.997 (3)	148
C11—H11A···O1	0.93	2.57	3.196 (3)	125
C12—H12A···O4^ii^	0.93	2.44	3.193 (3)	138
C13—H13A···O1^iii^	0.93	2.47	3.376 (3)	164
C10—H10A···Cg3	0.93	2.93	3.688 (2)	140

Symmetry codes:  (ii) −*x*+1, *y*, −*z*+1/2; (iii) *x*, −*y*+1, *z*+1/2.

## References

[bb1] Applegarth, L., Goetra, A. E. & Steed, J. W. (2005). *Chem. Commun.* **18**, 2405–2406. 10.1039/b419216h 15877143

[bb2] Enraf–Nonius (1989). *CAD-4 Software.* Enraf–Nonius, Delft, The Netherlands.

[bb3] Farrugia, L. J. (1999). *J. Appl. Cryst.* **32**, 837–838.

[bb4] Gabe, E. J., Le Page, Y., Charland, J.-P., Lee, F. L. & White, P. S. (1989). *J. Appl. Cryst.* **22**, 384–387.

[bb5] Meerssche, M., Germain, G., Declercq, J. P. & Touillaux, R. (1979). *Cryst. Struct. Commun.* **8**, 119–122.

[bb6] Meerssche, M., Germain, G., Declercq, J. P., Touillaux, R., Roberfroid, M. & Razzouk, C. (1980). *Cryst. Struct. Commun.* **9**, 515–518.

[bb7] North, A. C. T., Phillips, D. C. & Mathews, F. S. (1968). *Acta Cryst.* A**24**, 351–359.

[bb8] Sheldrick, G. M. (2008). *Acta Cryst.* A**64**, 112–122. 10.1107/S0108767307043930 18156677

